# Immunodiagnosis of Visceral Leishmaniasis: Current Status and Challenges: A Review Article

**Published:** 2018

**Authors:** Bahador SARKARI, Zahra REZAEI, Mehdi MOHEBALI

**Affiliations:** 1. Dept. of Parasitology and Mycology, School of Medicine, Shiraz University of Medical Sciences, Shiraz, Iran; 2. Basic Sciences in Infectious Diseases Research Center, Shiraz University of Medical Sciences, Shiraz, Iran; 3. Dept. of Medical Parasitology and Mycology, School of Public Health, Tehran University of Medical Sciences, Tehran, Iran

**Keywords:** Immunodiagnosis, Visceral leishmaniasis, Antibody detection, Antigen detection

## Abstract

**Background:**

Diagnosis of Visceral Leishmaniasis (VL) is still challenging. This review highlighted current status and challenges in the serological diagnosis of VL. Furthermore, the drawback of currently available serological tests and the most recent advancement in the designing and application of these assays for the diagnosis of VL are addressed.

**Methods:**

All the published literature cited within PubMed, ISI Web of Science, Google Scholar, Scopus, and IranMedex, regarding the immunodiagnosis of VL in human were sought from 2000 till Mar 2017. The search terms were “visceral leishmaniasis”, or “kala-azar” subsequently combined with the search terms “diagnosis”, “serodiagnosis”, “human”, “serological”, “antigen detection” or “antibody detection”. Data were extracted from literature which fulfilled our eligibility criteria.

**Results:**

Direct agglutination test (DAT) and rk39 dipstick have made a great improvement in the serological diagnosis of VL. Besides, other kinesin-related protein including K26, K28, and KE16 provided promisingly diagnostic accuracy in the diagnosis of VL. The Latex Agglutination Test for the diagnosis of VL (KAtex), with moderate sensitivity but high specificity, made a substantial contribution to the field. Moreover, a range of protein antigens has recently been detected in the urine of VL patients with encouraging diagnostic value.

**Conclusion:**

The suboptimal diagnostic accuracy of the currently available serological assays for the diagnosis of human VL necessitates further research and development in this field. Outcomes of immunodiagnostic tests based on recombinant antigens are favorable. These proteins might be the most appropriate antigens to be further evaluated and utilized for the diagnosis of human VL.

## Introduction

Visceral leishmaniasis (VL) continues to pose a significant health problem in several countries in tropical and subtropical areas of the world (1–5). In Iran, VL has been reported sporadically in all of 31 provinces of the country, but the disease is endemic in some parts of northwestern and southern areas with an annual rate of 100–300 new cases (5–8). In recent years, the annual number of symptomatic cases of human VL in Iran reached to 0.092 per 100,000 general populations (9).

The disease is fatal if left untreated in more than 95% of cases (10). Proper management of VL requires an accurate diagnosis. Therefore, highly sensitive and specific diagnostic tests are needed for appropriate diagnosis of the disease.

The gold standard of diagnosis of VL remains the detection of *Leishmania* amastigotes forms of the parasite in tissue samples. The sensitivity rates of this method varies from 50% to 99% based on the tissue from which the sample is prepared, being most sensitive for spleen (93%–99%), moderate for bone marrow (53%–86%) and least sensitive (<50%) for lymph nodes specimens (11). Besides, the method is invasive and may carry the fetal risk, in splenic aspiration, for the patients.

Molecular approaches are technically demanding and might not be an appropriate method for the diagnosis of VL, especially in countries with poor facilities and resources. The diagnostic accuracy of these methods for the diagnosis of VL, but not solely the parasite infection, in VL-endemic areas is questionable. A recent meta-analysis about molecular tools for diagnosis of VL revealed that pooled sensitivity of PCR in whole blood is 93.1% and its specificity is 95.6%. The specificity has been significantly lower (63.3%) in consecutive studies due to the number of the asymptomatic carriers in an endemic area (12–14).

An ideal test for serodiagnosis of VL would be a test which is cost-effective, easy-performing, with both high sensitivity and specificity. Currently, the main serodiagnostic assays for the diagnosis of VL are Direct Agglutination Test (DAT), Enzyme-linked Immunosorbent Assay (ELISA), Indirect Immunofluorescence Antibody Test (IFAT), dip-stick assays and the antigen-based latex agglutination test (KAtex) (11, 15–19).

This review summarizes the performances of the main serological diagnostic assays for the diagnosis of human VL and highlights the recent developments in this field, made over the last decade. The review also highlights the performances of different leishmanial antigens in the diagnosis of VL.

## Methods

All the published literature cited within PubMed, ISI Web of Science, Google Scholar, Scopus, and IranMedex, regarding the immunodiagnosis of VL in human, were sought from 2000 till Mar 2017. A few older published papers were also included in the review because of their originality. The search terms were “visceral leishmaniasis”, or “kala-azar” subsequently combined with the search terms “diagnosis”, “serodiagnosis”, “human”, “sero-logical”, “antigen detection” or “antibody detection”. A few studies were also found by back tracing the reference lists of the articles. Data were extracted from literature which fulfilled our eligibility criteria.

## Results

A variety of immunodiagnostic assays, using different antigens including whole parasite promastigotes or amastigotes, synthetic peptides and crude or recombinant antigens have been extensively used for the diagnosis of VL. These immunodiagnostic methods are either antibody or antigen-based assays.

### Antibody-based diagnostic assays

A range of antibody detection assays has been developed and evaluated in the field for the diagnosis of VL. The most studied ones are DAT, IFAT, ELISA and rapid immunochromatographic tests. Their main drawbacks are positivity long after cure and also positivity in asymptomatic cases in the VL-endemic areas. Therefore, antibody-based assays must always be interpreted in light of clinical pictures of the patient.

### Direct agglutination test (DAT)

DAT was adopted for the diagnosis of VL by El-Harith (20). The test is currently being used for the diagnosis of VL in a few VL-endemic countries. DAT can be performed on serum, plasma or even urine (17, 21). The test can be performed on urine samples with diagnostic accuracy somewhat similar to the sera samples (18, 21, 22). The performance of DAT is neither *Leishmania* species-specific nor region dependent. Among the validated sero-logical tests, DAT was found to be more specific (specificity rates of 72%–100%), sensitive (sensitivity rates of 92%–100%) and practically applicable test (23, 24). A meta-analysis, comparing DAT and rK39 strip test, found that DAT is almost 1% more sensitive and 2% more specific than rK39 strip test (25).

DAT in its freeze-dried form facilitated its use in the field (26–29). A freeze-dried antigen of *L. infantum* in DAT demonstrated 99% sensitivity and 98.2% specificity, which was similar to the conventional DAT when the test carried out on 103 sera of VL patients in Brazil (30). Major shortcomings of DAT are multiple pipetting, long incubation time, batch to batch variability of the antigen and cross-reactivity with other trypanosomatids.

DAT titer decline over time falling below the cut off (1:800) at the ninth month of cure but still remains positive for relatively long time (up to 5 yr in more than 50% of VL cases) after the cure. Thus, the test cannot be used for follow up of treatment or for the diagnosis of disease relapse (31). To overcome the problem of long incubation time, a fast agglutination screening test has been introduced, which uses only one serum dilution and require only three hours of incubation (32). The validity of fast DAT for the detection of *L. infantum* infection in the field was compared with the conventional DAT in Iran and the results showed a sensitivity of 95.4% and specificity of 88.5% for fast DAT in comparison with conventional DAT (33).

### Immunofluorescence antibody test (IFAT)

IFAT is based on detection of antibodies against promastigotes or amastigotes forms of the parasite. IFAT was evaluated for the diagnosis of VL in Iran and the authors concluded that this test is the most practical and reliable assay for the laboratory diagnosis of VL (34). In other studies, IFAT has shown a sensitivity of 87%–100% and specificity of 77%–100% for the diagnosis of VL (34, 35). Cross-reactivity can be seen in patients with tuberculosis, toxoplasmosis, malaria, typhoid fever, or brucellosis in IFAT (21, 36). A comprehensive systematic review and meta-analysis about the diagnostic performance of serological tests for the diagnosis of VL found a poor sensitivity (11%–82%) with higher specificity (79%–100%) for IFAT (37).

### Enzyme-linked immunosorbent assay (ELISA)

A variety of antigens have been adopted in ELISA system for the diagnosis of VL with different sensitivity and specificity. Cross-reactivity with sera of toxoplasmosis, tuberculosis, and trypanosomiasis has been reported with *Leishmania* crude antigen (17, 21).

### Rk39-based antibody detection assays

RK39 derived from a 39 amino acid repeat encoded by a kinesin-like protein-encoding gene of *Leishmania chagasi*. Development of rK39 dipstick test brought a significant improvement in the serological diagnosis of VL in the field. A systematic review with meta-analysis of the studies published in the literature regarding the performance of rK39 in comparison with other serological assays found that rK39, either in strip or ELISA format, along with DAT had the best performance for the serodiagnosis of VL (38).

Another meta-analysis showed sensitivity estimate of 93.9% and specificity of 93.6% for rK39 and sensitivity and specificity of 94.8% and 85.9% for DAT (39). The rK39-based dipstick had lower sensitivity in Sudan in comparison with the Indian subcontinent. Specific polymorphisms have been found between coding sequences of rK39 homologous from South Asian and East African strains. These differences in genetic diversity influence the performance of rK39 diagnostic assays in these two regions (40). However, a new version of the strip test (DiaMed AG, Cressier Sur Morat, Switzerland) showed satisfactory results in Sudan (41).

RK39 has been used to detect anti-*Leishmania* antibodies in saliva, but the results were not satisfactory (42). The rK39 strip test has also been used for the diagnosis of VL in human urine. The test showed 96.1% sensitivity and 100% specificity (43). In another study, rK39 was used for the detection of VL by using urine samples with 100% sensitivity and 86% specificity. However, urine-based testing had more false-positive results than blood testing (44).

The negative predictive value (NPV) of the rK39 test in East Africa was found to be 81% as compared to 95% in the Indian sub-continent (45). In one study in Ethiopia, the NPV of rK39-ICT was 41.7%. This indicates that rK39-ICT negative test does not necessarily rule out the presence of VL in a patient (46). The positive predictive values (PPV) of rK39-ICT have been reported to be 69% up to 99.1% in different studies (25, 46–49). Lower PPV was reported from Ethiopia (74% and 69.6%) while higher PPV were reported from Sudan (81%), Brazil (99.1%), Kenya (92.9%) and Bangladesh (98.3%) (46–49). rK39-ICT positivity does not necessarily indicate the presence of active VL in significant portion of individuals.

### Antibody detection based on RkE16, rK26, rK28 K9 and KRP42 antigens

Other kinesin-related proteins including K9, K26, rK28, KRP42, and KE16 have been evaluated for the diagnosis of VL with variable diagnostic accuracy (50–56). Evaluation of two prototype lateral flow-based rK28 rapid tests on VL patients provided high sensitivities in Sudan (95.9%) and in Bangladesh (98.1%) compared to the rK39 RDT (57).

Reasonable sensitivity and specificity (99.6% and 94.1%–100%, respectively) were obtained with an ELISA system, utilizing an rK28 fusion protein for the detection of anti-*Leishmania* antibodies in India (55). RK39 and rK28 antigens have similar sensitivity and specificity in the diagnosis of VL (55, 57).

In Sudan, a recombinant antigen of *L. donovani* (rKLO8) which contained putative conserved domains with significant similarity to the rK39 and KE16 showed higher sensitivity (98.1%) and specificity (96.1%) in comparison with rK39 in an ELISA system (58). Combination of rK39 and rK26 has been used as a rapid diagnostic test with satisfactory sensitivity (100%) and specificity (98.9%). A latex agglutination test using A2 antigen of amastigotes of *L. infantum* had a sensitivity and specificity of 88.4% and 93.5% respectively when tested on sera samples of VL patients and controls in Iran (59). Of the non-protein antigens, 9-O-acetylated sialic acid has been shown to have potential serodiagnostic value for the diagnosis of VL (60).

Diagnostic performance of crude *Leishmania* histone (CLH) in an ELISA system was evaluated in Mediterranean VL patients with 97.6% sensitivity and 100% specificity, a diagnostic performance similar to rK39-based ELISA (61).

### Antigen-based diagnostic assays

Antigen detection has been used for the diagnosis of several parasitic diseases with encouraging performance (17, 19, 62). For VL, the most studied antigen-based assay is the KAtex which detects a low molecular weight carbohydrate antigen in the urine of VL patients (63, 64).

### KAtex

KAtex was originally developed by Attar at Liverpool School of Tropical Medicine, UK, in 2001 (64). The test was commercialized by Kalon Biological Ltd (Guildford, UK), known as KAtex. KAtex detects only active disease and quickly turn negative after successful treatment. The test gives a result in a few min. To eliminate the cross-reacting substances, urine sample needs to be boiled (by immersing briefly in boiling water) and cooled to ambient temperature before testing (65). Attempt has been made to improve the KAtex by removing the unpleasant boiling process but with limited success (66).

The test has been evaluated in different VL-endemic areas with high specificities (80%–100%) and moderate sensitivity (40%–95%). KAtex showed 79.1%–94.1% specificity and 60.4%–71.6% sensitivity in India (15). KAtex showed a sensitivity and specificity of 71% and 64% in Ethiopia, 84% and 87.8% in Kenya, 72.9% and 98.3% in Sudan, 66.1% and 87.6% in India, 35.8% and 97.8% in Nepal, and, 82.7% and 98.9% in Iran (15, 67). A monoclonal antibody has been raised against the KAtex target antigen and used in a capture ELISA with a sensitivity of 94.1% and specificity of 100% for the diagnosis of VL (62).

The PPV of KAtex is usually high, ranged from 74% to 99% while the NPV is lower, ranging from 35% to 99% (19, 25, 68, 69). The PPV and NPV for KAtex have been reported to be 98% and 56% respectively when VL suspected patients and healthy controls were evaluated in rural areas of Nepal (25). Being a promising test, the urine-based KAtex suffer from low sensitivity and this inherited shortcoming needs to be improved.

### Assays for detection of leishmanial Urinary polypeptides

The sensitivity and specificity of the 72–75 kDa protein, detected in the urine of VL patients, have been reported to be 96% and 100%, respectively (70). A urinary antigen detection assay, developed for the detection of *L. infantum* iron superoxidase 1 and nuclear transport factor 2, had a sensitivity of 89% and specificity of 100% (71). The assay is based on the detection of only *L. infantum* antigen and may not properly detect the antigen of *L. donovani*, which is the main causative agent of VL in the Old World (72). Table 1 shows the performance of different antigens detection assays in the diagnosis of VL.

**Table 1:** Performance of different antigen detection systems in the diagnosis of human VL

***Sample***	***Test***	***No. of subjects***			***Sensitivity (%)***	***Specificity (%)***	***Country***	***Ref.***
		**VL patients**	**Other diseases**	**Healthy control**				
Urine	KAtex	25	34	312	68	100	Brazil	(64)
Urine	KAtex	29	23	312	100	100	Yemen	(64)
Urine	KAtex	5	-	312	86	100	Nepal	(64)
Urine	KAtex	62	16	158	95.2	100	Sudan	(73)
Urine	KAtex	155	77	-	47.7	98.7	Nepal	(74)
Urine	KAtex	382	-	185	NR	99	India	(75)
Urine	KAtex	85	57	-	57	98	Nepal	(25)
Urine	KAtex	63	-	38	57.4	84.3	Ethiopia	(68)
Urine	KAtex	282	70	100	67	99	India	(76)
Urine	KAtex	33	-	35	100	91.4	Bangladesh	(77)
Urine	KAtex	313	14	57	77.7	98.2	Iran	(78)
Urine	KAtex	38	-	-	71	64	Ethiopia	(15)
Urine	KAtex	308	-	-	84.5	87.8	Kenya	(15)
Urine	KAtex	294	-	-	72.9	98.3	Sudan	(15)
Urine	KAtex	352	-	-	66.1	87.6	India	(15)
Urine	KAtex	158	-	-	35.8	97.8	Nepal	(15)
Urine	KAtex	50	-	50	94	98	Bangladesh	(79)
Urine	KAtex	36	-	40	75	100	Bangladesh	(80)
Urine	KAtex	46	30	46	61	92	Ethiopia	(81)
Urine	KAtex	13	30	46	69	92	Bangladesh	(81)
Urine	KAtex	43	30	46	56	92	Brazil	(81)
Urine	KAtex	100	-	50	87	100	Bangladesh	(69)
Urine	Antigen™ Detect ELISA	46	30	46	93.5	100	Ethiopia	(81)
Urine	Antigen™ Detect ELISA	64	30	46	96.9	100	Sudan	(81)
Urine	Antigen™ Detect ELISA	13	30	46	100	100	Bangladesh	(81)
Urine	Antigen™ Detect ELISA	43	30	46	88.4	100	Brazil	(81)
Urine	Capture ELISA	35	24	10	60	91.2	Iran	(17)
Urine	Capture ELISA	34	-	104	94.1	100	Yemen, Nepal, Spain, Sudan, Brazil	(62)

### Serological tests for the diagnosis of HIV/VL co-infection

HIV/VL co-infected cases can be properly diagnosed with up to 98% sensitivity if DAT and rK39 are used together (82). Findings of a comprehensive meta-analysis on serological diagnosis of VL revealed that serological tests should not be used in ruling out of VL infection in HIV/VL co-infected subjects (37). In a recent meta-analysis study the diagnostic accuracy of IFAT, ELISA, immunoblotting, and DAT were evaluated for the diagnosis of VL in HIV/VL co-infected patients and concluded that serological tests have inclusive limited sensitivity for the diagnosis of VL in HIV/VL cases (83). The overall sensitivities of the tests were 84% for immunoblotting, 81% for DAT, and 66% for ELISA, while their specificities were 82% for immunoblotting, 90% for ELISA, 93% for IFAT and 90% for DAT (83). Among the serological tests, DAT and immunoblotting have better global accuracy for the diagnosis of VL in HIV/VL co-infection (83). In a study on HIV/VL patients in Brazil, IFAT and rK39 dipstick tests exhibited the lowest sensitivity while DAT demonstrated a good overall performance for the diagnosis of VL in HIV/VL patients (84). All of the DAT seropositive cases had clinical signs and symptoms of VL, when DAT was used for the diagnosis of VL in HIV/AIDS cases (85). Comparison of IFAT and DAT for serodiagnosis of VL in HIV-infected subjects showed that these two tests have a similar specificity but that IFAT has a higher sensitivity (86). The lower sensitivity of DAT (89%) was reported in HIV/VL co-infected patients in comparison with HIV-negative patients (95%) (87).

Antigen detection might be a valuable approach for the diagnosis of HIV/VL co-infection, as the level of antibodies in these patients is low. Sensitivity of 85.7% and specificity of 100% were reported when KAtex was used for the diagnosis of VL in HIV/VL patients in Spain (88). KAtex was used for the diagnosis of VL in urine samples of HIV/VL patients from Spain with 90% sensitivity and 100% specificity (Sarkari et al., unpublished data).

## Discussion

Much endeavor has been devoted over the last two decades to develop a field applicable, easy-to-perform, and accurate diagnostic test for the diagnosis of VL. Among the developed antibody detection assays, DAT and rk39 dipstick tests have made a great improvement in the serological diagnosis of VL. When evaluating the diagnostic accuracy of an immunodiagnostic test, one should always consider the PPV and NPV values. These features are clinically valuable and may influence therapeutic decisions by the physicians. Positive and negative predictive values of any immunodiagnostic test are directly connected to the prevalence of the disease.

The NPV of the rK39 test is low in few of VL-endemic areas. The test does not necessarily rule out the VL infection. On the other hand, the PPV of rK39-based assays has been low in areas such as Ethiopia. This, in turn, shows that rK39-ICT positivity does not necessarily indicate the presence of active VL in a significant portion of individuals in the VL-endemic areas.

KAtex with its moderate sensitivity and high specificity has made a substantial contribution to the diagnosis of VL. In line with this, a range of protein antigens has recently been detected in the urine of VL patients with reassuring diagnostic value.

Perhaps one of the greatest challenges in the serological diagnosis of VL is the discrepancies related to the results obtained from relatively similar serological assays in different VL-endemic areas. These inconsistencies are likely due to the method of antigen preparation, testing methods, purity of employed antigen, the strain of *Leishmania* and characteristics of the evaluated subjects. The other obstacle in the serological diagnosis of VL is that the main antibody detection assays are positive in asymptomatic cases in VL-endemic areas and that the tests remain positive long after the successful treatment (89). Hence, they cannot differentiate between past and present infections. Bearing this in mind, results of immunodiagnostic tests have to be interpreted in light of the clinical presentation.

## Conclusion

The suboptimal diagnostic accuracy of the currently available serological assays for the diagnosis of human VL necessitates further research and development in this field. Performances of the immunodiagnostic tests based on recombinant proteins are satisfactory. These proteins might be the most appropriate antigens to be further evaluated and utilized for the diagnosis of human VL. A new perspective in the development of serological assays for the diagnosis of VL might be the utilization of fused protein, through combining of several well-defined antigens. Moreover, the detection of polypeptide urinary antigen seems to be an encouraging mission forward carried out in the future for the development of a suitable antigen-based assay for the diagnosis of VL.
